# Wing Shape and Its Influence on the Outcome of Territorial Contests in the Damselfly *Calopteryx virgo*

**DOI:** 10.1673/031.012.9601

**Published:** 2012-08-12

**Authors:** Jessica Bots, Casper J. Breuker, Kari M. Kaunisto, Jani Koskimäki, Hans Van Gossum, Jukka Suhonen

**Affiliations:** ^1^ Evolutionary Ecology Group, University of Antwerp, Antwerp, Belgium; ^2^ Evolutionary Developmental Biology Research Group, Faculty of Health and Life Sciences, Department of Biological and Medical Sciences, Oxford Brookes University, Oxford, UK; ^3^ Section of Ecology, Department of Biology, University of Turku, Turku, Finland; ^4^ University of Oulu, Evolution and Behaviour Research Group, Oulu, Finland

**Keywords:** aspect ratio, geometric morphometrics, male-male competition, resource holding potential, wing design

## Abstract

Male mating success is often determined by territory ownership and traits associated with successful territory defense. Empirical studies have shown that the territory owner wins the majority of fights with challenging males. Several physical and physiological traits have been found to correlate with resource holding potential. In addition, in aerial insects, wing design may also have a strong influence on resource holding potential, since it determines efficiency and precision during flight. However, this possibility has not yet been thoroughly evaluated using the modern technique of geometric morphometrics to analyze shape. Therefore, this study examined whether wing shape affects the outcome of male-male contests in the territorial damselfly, *Calopteryx virgo* (L.) (Odonata: Calopterygidae). Wing shape and also traditional flight-related morphological measures were compared between 27 pairs of winners and losers from experimental territorial contests. Contrary to expectations, there were no differences between winners and losers in all studied wing traits (shape, length, width, total surface, aspect ratio, and wing loading). However, highly significant differences in wing shape and size were detected between the fore- and hindwing. It is currently not known how these differences relate to flight performance, since previous biomechanical studies in damselflies assumed fore- and hindwings to have an identical planform.

## Introduction

Territorial behavior is commonly observed in animal mating systems and is known to have evolved many times independently (Andersson 1994). This indicates that the benefits obtained from this behavior exceed the costs of fighting for mating opportunities, such that territorial behavior and traits associated with successful territory defense will likely be favored by natural (Suhonen et al. 2008) and sexual selection (Hunt et al. 2009). Recent reviews show that male-male territorial contests have received much interest by behavioral and evolutionary biologists (Kemp and Wiklund 2001; Kokko et al. 2006; Briffa and Sneddon 2007; Suhonen et al. 2008). Much knowledge of territorial behavior has been obtained from theoretical studies modeling the evolution of fighting strategies and from empirical examination of phenotypic traits that affect contest outcome (reviewed in, e.g., Kokko et al. 2006; Briffa and Sneddon 2007).

In many of the empirical studies, it has been demonstrated that the territory owner wins the majority of fights with challenging males (e.g., Davies 1978; Waage 1988; Switzer 2004). Current explanations for resident advantage include that individuals are following conventional rules, such as ‘respect for ownership’ (Kokko et al. 2006). Also, prior differences may exist between owners and intruders in resource holding potential (RHP, i.e., some feature that enhances fighting ability) or the value of the territory (e.g., Kemp and Wiklund 2001; Switzer 2004). Strong support for a prior-residence effect has been found when: (1) individuals are more or less matched in size, (2) when individuals with high RHP tend to accumulate as owners, and (3) when ownership allows a higher RHP (e.g., a sunspot that provides an insect resident with a thermoregulation advantage (Stutt and Willmer 1998)) (reviewed in Kokko et al. 2006). To examine the origin of the asymmetries in RHP between territory owners and intruders, many studies have used Odonates as a model system (Suhonen et al. 2008). From these studies, it is known that several physical and physiological characteristics of contestants may correlate with RHP, including age, ornament size, fat reserves, and immunocompetence (e.g., Marden 1989; Marden and Waage 1990; Marden and Rollins 1994; Plaistow and SivaJothy 1996; Koskimäki et al. 2004; Suhonen et al. 2008). By contrast, body size, wing aspect ratio, flight muscle mass, or muscle power output seldom affect RHP in Odonates (reviewed in Suhonen et al. 2008; but see, e.g., Moore 1990).

**Figure 1.**
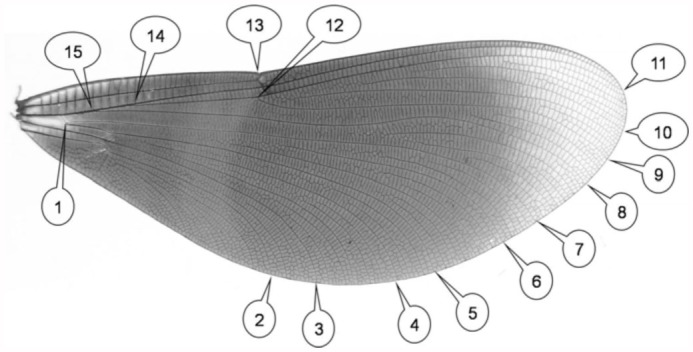
Landmarks that were digitized on the homologous fore-and hindwing of *Calopteryx virgo*. High quality figures are available online.

In addition, wing design may correlate with RHP in aerial insects, including Odonates. It has been shown in the territorial damselfly *Lestis viridis* that flight-related morphology affects mating success (De Block and Stoks 2007; Swillen et al. 2009). This suggests that maneuverability and agility during flight may help males acquire mating sites and defend them successfully in territorial contests. In general, to favor high maneuverability, wings should be short and broad, while wings that are long and slender enhance lift production (Betts and Wootton 1988). However, it is not known whether such variation in wing shape among males is related to their potential of winning territorial fights. Previous studies seem to suggest an absence of a relationship between RHP and the flight apparatus. However, these studies have only considered single aspects of wing morphology (reviewed in Suhonen et al. 2008), such as aspect ratio, and thus have only investigated univariate traits. Wing shape is a multivariate trait and thus the effects of wing shape should be examined using geometric morphometrics. Geometric morphometrics provides a detailed multivariate description of shape by incorporating information from well-chosen landmarks across the wing (Zelditch et al. 2004), which allows detecting more subtle variation compared to aspect ratio. For instance, since aspect ratio is determined as the squared wing length divided by the surface area, it is possible to have similar results for structures that have an identical surface area but a different shape (e.g., a damselfly wing with the widest midline positioned more anterior; see Figure 1). In agreement, a recent study in Odonates showed that wing shape is related to mating behavior, but similar differences could not be detected using the cruder measure, aspect ratio (Johansson et al. 2009).

In general, wing design is known to have a strong effect on many aspects of flight performance of insects including maneuverability, agility, lift, and thrust production (e.g., Dudley 2000; Berwaerts et al. 2002), and hence may ultimately affect fitness (Kölliker-Ott et al. 2003). Much of the reported within-species variation in wing morphology has therefore been interpreted as an adaptive response to varying environmental conditions (Pètavy et al. 1997; Azevedo et al. 1998, Van Dyck and Wiklund 2002) or different behavioral strategies (Breuker et al. 2007). Such within-species variation in wing morphology occurs mostly between males and females (e.g., Van Dyck and Wiklund 2002; Breuker et al. 2007), but can also be pronounced between members of the same sex (Van Dyck and Wiklund, 2002; Bots et al. 2009). For instance, in the butterfly *Pararge aegeria*, males locate females by territorial perching in sunlit patches or by patrolling through the forest (Shreeve 1987). Wing design of late-spring males has more characteristics favoring territorial perching (i.e., larger relative thorax mass, wing loading, and aspect ratio) than that of summer males, which is in agreement with the value of a territory being higher under cooler conditions (Van Dyck and Wiklund 2002). Similarly, variation in wing design may occur among territorial males in Odonates and may influence their RHP, but this hypothesis requires further investigation.

This study tests whether the outcome of male-male territorial contests can be predicted by wing shape in the territorial damselfly *Calopteryx virgo* (L.) (Odonata: Calopterygidae). *Calopteryx* males settle territory ownership by aerial contests that can become highly escalated and prolonged between closely matched contestants (Marden and Waage 1990; Marden and Rollins 1994; see also Fitzstephens and Getty 2000). The outcome of the contests has been shown to be determined by differences in stored energy, with winners having significantly more fat reserves after the contest than losers (Marden and Waage 1990; Plaistow and Siva-Jothy 1996; Koskimäki et al. 2004). However, it has not been considered whether the difference in energy expenditure between winners and losers may be affected by variation in (the aerodynamic properties of) wing design (but see Marden and Waage 1990). To test our hypothesis, experimental contests were staged between *C. virgo* males. The wing shape of winners and losers was examined using geometric morphometrics. Results were also compared with traditional measures of wing morphology routinely reported in the literature.

## Materials and Methods

### Study species

*Calopteryx virgo* is a sexually dimorphic insect species that lives along small rivers and streams with moderate flow and some submerged vegetation (Pajunen 1966; Rantala et al. 2001). Most of *C. virgo* males defend well-defined territories, consisting of patches of floating vegetation, which females use as oviposition sites. These territorial males are stationary and may occupy the same territory for up to several weeks (Corbet 1999; Córdoba-Aguilar and Cordero-Rivera 2005). They normally expel intruders with brief pursuit flights, but occasionally encounters may last up to an hour or more (Koskimäki et al. 2004). In contrast, non-territorial males perch higher in the vegetation, change perch frequently, and spend much of their time flying along the river (Pajunen 1966; Koskimäki et al. 2009).

### Experimental territorial contests

Experimental contests between territorial males were performed between 2 and 15 July 1999 at Mustajoki Creek and Vispiläjoki Creek, near the city of Jyväskylä (62° 16′ N, 25° 30′ E) in central Finland (see also Koskimäki et al. 2004). All experiments were carried out between 10:00 and 18:00, when the damselflies were reproductively active. Males were aged by assigning them to one of four age categories defined by the stiffness of the leading edge of the wings, which increases with age (see Plaistow and Siva-Jothy 1996). The age categories ranged from class 0, which were freshly emerged individuals, to class 4, which were damselflies nearing the end of their lives characterized by hard and broken wings. All males used in contests belonged to age class 2, which are males with hard but undamaged wings (Plaistow and Siva-Jothy 1996). Also, all males were individually marked on the hindwing with an enamel pen prior to the experiment (see Plaistow and Siva-Jothy 1996; Rantala et al. 2001). Contests were staged between neighboring males by gradually merging artificial territories consisting of clumps of floating vegetation (see Waage 1988; Marden and Waage 1990; Koskimäki et al. 2004). Merging territories causes both males to act as residents of the same territory, thereby removing the normal resident-intruder asymmetry (Waage 1988). Consequently, every contest in this study determined a chance in residency, and allowed to quantify the morphological characteristics of winners and losers. After the contest, winners were defined as the individual that perched in the territory while the loser fled. In total, 10 contests were staged at Mustajoki Creek and 17 at Vispiläjoki Creek, which resulted in a total of 27 winners and 27 losers that were collected. The contest duration varied widely from 0.5 to 83 min, and was on average 33 min (SD = 35). After each experimental contest, the collected damselflies were stored in individual black plastic containers inside a cold box and were taken to the laboratory for further measurements (for information on fat reserves determined for the same set of individuals see Koskimäki et al. 2004).

### Wing morphology

To quantify differences in wing morphology between the winners and losers, both the multivariate trait of wing shape, and the traditional measures of wing morphology such as aspect ratio, length, width, total surface, and wing loading of both fore- and the hindwings were determined. The wings from each individual were carefully removed with two pairs of fine forceps. Each wing was then placed between two microscope slides to ensure that it was flat prior to imaging. Fore-and hindwings were subsequently photographed with a digital camera (following the routine described in Breuker et al. 2007). Using these digital pictures, 15 landmarks (i.e., 30 coordinates, Figure 1) were digitized on the homologous fore- and hindwings in ImageJ 1.38 (Abramoff et al. 2004). Landmarks studied were located either where wing veins meet the wing edge or at vein intersections in the central area of the wing (Figure 1), and thus provide a measure of overall wing shape. One individual had damaged wings due to handling and was excluded from all analyses (thus data of its challenger were also excluded in paired comparisons, see below). To evaluate the effect of measurement error, both repeat photos and measurements were taken and a Procrustes ANOVA was performed (Klingenberg and McIntyre 1998). The mean squares for individual shape variation and the asymmetry variation (i.e., individual* side interaction) were significantly larger than the error due to imaging and measuring the images (*p* « 0.001), thus confirming that measurement error was negligible compared to biological shape and size variation.

Variation in wing shape was examined using generalized least squares Procrustes superimposition methods, which consists of four steps: (1) reflection to either left or right configuration, (2) scaling of configurations to unit centroid size, (3) superimposing the centroid of the configuration to the centroid of the consensus configuration, and (4) rotation around the centroid to obtain optimal alignment (e.g., Goodall 1991; Klingenberg and McIntyre 1998). Procrustes superimposition therefore removes variation in reflection, translation, scaling, and rotation, but preserves shape. The analysis yielded Procrustes coordinates based on the averaged values of left and right wings. Using these Procrustes coordinates of fore- and hindwing, principal component analyses (PCA) were carried out in MorphoJ (Klingenberg 2011). For the forewing, PC1, PC2, and PC3 respectively explained 51.3, 13.4, and 8.4% of the variation in wing shape, whereas these PC values for the hindwing explained respectively 46.6, 14.0, 8.1%. To evaluate differences in wing shape between winners and losers, variation in PC1, PC2, and PC3 was analyzed jointly in R (cran.r-project.org) using a paired Hotelling's T^2^-test, which is the multivariate equivalent of a paired *t*-test (Zelditch et al. 2004). The analyses were carried out separately for the fore- and hindwing, since these differed significantly in wing shape (see below). Comparison of the fore- and hindwing shape was done in SAS version 9.1 (www.sas.com) using a mixed model ANOVA with PC values obtained from PCA based on the combined dataset of Procrustes coordinates. PC1 and PC2 were used as dependent variables, which respectively explained 64.8 and 12.0 % of the shape variation. PC3 was not included in this analysis since it only explained 5.0 % of the variation. Also, since wings collected from the same individual are not independent, individual was added as a random factor to the model.

In addition, variation in the slenderness of the wings of winners and losers was evaluated by determining the aspect ratio ((2^*^ mean wing length)^2^/ mean wing surface) of the (averaged values of left and right) fore- and hindwings. Wing surface was calculated using the original landmark coordinates according to Green's Theorem (Kaplan 1991), whereas wing length was determined as the distance between landmark 1 and 11 (Figure 1).

Furthermore, wing loading was determined by dividing body mass by total wing surface (body mass / (2*(mean forewing surface + mean hindwing surface))). Fresh mass was measured (after the contest) to the nearest 0.1 mg with a Ham-bascgb-1 electronic balance (A and D Instruments, www.aandd-eu.net). Total wing surface, and therefore also wing loading, could not be determined for two additional individuals for whom it was not possible to determine landmark coordinates of both the fore- and the hindwing due to damage. Finally, wing width was calculated as the distance between landmark 2 and 13 (Figure 1).

All analyses of the traditional measures of wing morphology were performed using data based on the average values of both left and right wings. Differences between winners and losers in aspect ratio, length, width, wing surface, body mass, and wing loading were analysed using a mixed model ANOVA in SAS version 9.1. “Wing” (fore- or hindwing) and “win” (winner or loser) were added as categorical factors to the statistical model. Also, the interaction “wing*win” was added to evaluate for potential different effects between fore- and hindwings. To account for the pairedness in the dataset (i.e., winners and losers tested in same contest), individual was nested within contest and treated as random factor. Also, site (Mustajoki Creek or Vispiläjoki Creek) was included as random factor. We started with the full model, including all main effects and interactions and proceeded with the removal of non-significant terms. The final model was obtained when only significant terms remained (Verbeke and Molenberghs 1997). Descriptive statistics are reported as mean ±SD.

**Figure 2.**
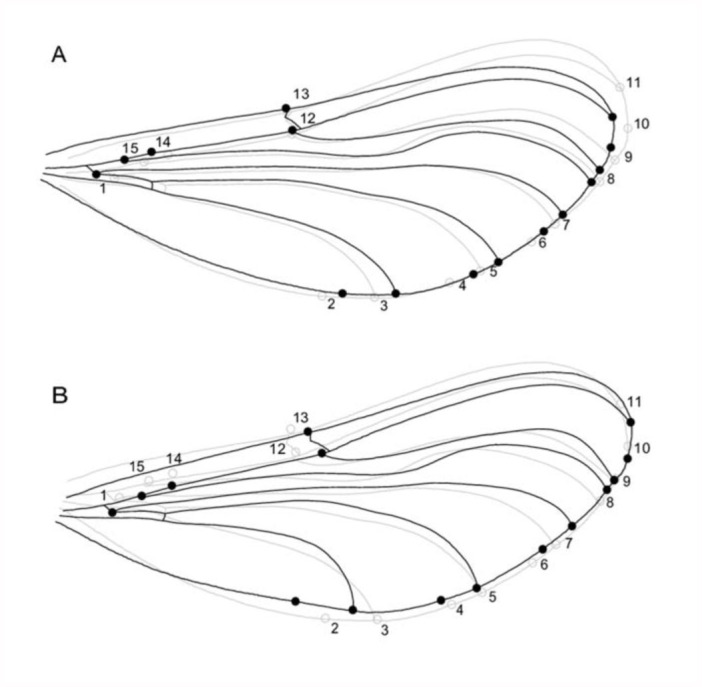
Warped outline drawings illustrate the shape changes associated with (A) PC1 and (B) PC2 (black outlines, solid dots) compared to the overall mean shape (gray outline, open dots). Changes are scaled to 0.1 Procrustus distance in the positive direction. PC1 primarily indicates variation in the curvature of the wing tip, whereas PC2 suggests variation in slenderness of the wing base between the fore- and hindwing. For clarity, only the main wing veins are depicted. High quality figures are available online.

## Results

**Figure 3.**
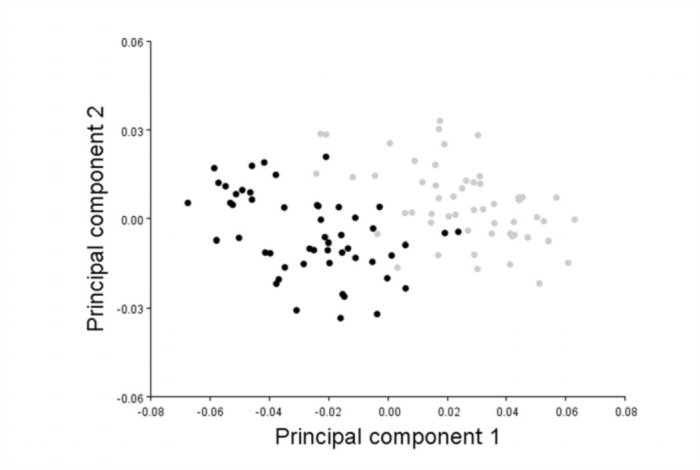
A scatter plot of PC1 versus PC2 shows that the fore-(gray dots) and the hindwing (black dots) can be almost completely separated based on these PC scores. High quality figures are available online.

No significant differences were detected in wing shape between winners and losers (forewing: T^2^ = 1.61, *F* = 0.49, *p* = 0.689; hindwing T^2^ = 3.13, *F* = 0.96, *p* = 0.431). By contrast, the fore- and hindwing of *C. virgo* males differed significantly in shape (PC1: *F*_1,52_ = 340.7, *p* < 0.01; PC2: *F*_1,52_ = 58.9, *p* < 0.01, Figure 2a, 2b). PC1 was primarily associated with changes in the curvature of the tip of the wing which was driven by variation in the position of landmarks 9, 10, and 11. The wing tip became less pointed with increasingly positive PC1 scores, while the opposite was true for negative values (Figure 2a). The change in PC2 primarily affected the slenderness of the wing base with landmarks 1, 2, 3, 14, and 15 moving towards the center of the wing. The wing base became narrower with increasingly positive PC2 scores while the opposite was true for negative values (Figure 2b). A scatter plot of PC1 against PC2 shows that fore- and hindwings can be almost completely separated based on these PC scores, with PC1 and PC2 scores being on average positive for the forewing and negative for the hindwing (Figure 3). Therefore, the forewing was on average less pointed at the wing tip and narrower at the wing base than the hindwing (Figures 2a, 2b, 3).

Results for the other measures of wing morphology were largely comparable to those of wing shape. Again, no differences could be detected in aspect ratio, wing length, width, surface or wing loading between winners and losers of territorial contests (Table 1). By contrast, the hindwing differed significantly from the forewing in aspect ratio, wing length, width, and surface (Table 1). Specifically, forewings were longer and wider than hindwings. Forewings also had a higher aspect ratio (Table 2), which indicates long and slender wings (*cfr*. shape analysis above). All raw data for fore- and hindwing of each individual sampled can be obtained from the Supplementary Table.

## Discussion

In this study, it was examined whether traits known to affect the flight performance in Odonates and other winged insects (e.g., Marden 1987; Betts and Wootton 1988; Dudley 2000), differed between winners and losers of territorial contests in the damselfly *C. virgo*. However, no differences were found using the traditional measures of wing morphology (including wing length, surface, loading, and aspect ratio), which confirms findings in previous studies (e.g., Marden and Waage 1990; Marden and Rollins 1994; Kemp 2000; Switzer 2004). Differences were also absent using the multivariate measure of wing shape, which was contrary to expectations. By contrast, it has been shown that males with longer wings more often occupy high quality territories (Tsubaki and Ono 1987). In our study, the value of the territory could not be taken into account since male-male fights were experimentally staged by merging artificial territories (Marden and Waage 1990). Hence, it cannot be excluded that wing shape or another flight-related trait affects the ability of a male to occupy a territory of higher quality. Such an effect may have important fitness consequences, since it is generally known that male mating success increases with territory size and quality (e.g., Andersson 1994). To evaluate this possibility, further studies are needed that compare wing morphology between territorial and nonterritorial males under natural conditions and relate it to the quality of the territory.

Results of this study thus do not alter the current viewpoint that resource holding potential (RHP) of *Calopteryx* males is predominately determined by physiological characteristics (reviewed in Suhonen et al. 2008). For the same *C. virgo* individuals we studied here for wing shape, it has previously been shown that winners have larger fat reserves and a higher immunocompetence, measured as the encapsulation response to a nylon monofilament (Koskimäki et al. 2004). In general, it has been demonstrated several times that both fat reserves and immunocompetence have a positive effect on the potential of winning territorial fights in *Calopteryx* damselflies (e.g., Marden and Waage 1990; Marden and Rollins 1994; Plaistow and Siva-Jothy 1996; Koskimäki et al. 2004). By contrast, the effects of flightrelated characteristics are probably nonexistent or at most limited.

While there seems to be little or no evidence that wing morphology plays a role in RHP, it has previously been shown that several wing characteristics affect predation probabilities (Svensson and Friberg 2007; Rantala et al. 2011). Specifically for *C. virgo*, stabilizing selection has been detected on both wing width and wing length such that males with intermediate values for both traits had a better chance of surviving predatory attacks by white wagtails (*Motacilla alba*) (Svensson and Friberg 2007). An important note, however, is that Svensson and Friberg (2007) collected wings after damselflies had been predated such that no distinction could be made between fore- and hindwings. This approach might have influenced results, certainly considering that we found forewings to be significantly larger, and to differ in shape from hindwings. For example, potential biases might result from variation in the relative proportions of fore- and hindwings that could be retrieved. This may explain the discrepancy with the study of Rantala et al. (2011), where no evidence for selection on wing size could be found when relating predation risk to hindwing length.

Shape variation between fore- and hindwings is common in Odonata. Indeed, in the larger and fast-flying Anisoptera forewings differ in planform from hindwings, but wings have been reported to be almost identical in Zygoptera (which include the Calopterigidae) (Wakeling 1997; Dudley 2000). By contrast, in this and another recent study (Outomuro and Johansson 2011), clear differences in shape were detected between the fore- and the hindwings using the more robust technique of geometric morphometrics. Differences in wing morphology among Odonates have previously been explained from the fact that in dragonflies an evenly distributed wing area is beneficial for fast flight, but that forewing evolution towards a broader wing base has been prevented by physical interference with the hindwing (Wakeling 1997). On the other hand, the absence of differences in damselfly wings is thought to be an adaptation to perform “clap and fling” flights, which requires a narrow base in both fore- and hindwings because of a small wing base separation (Wakeling 1997; Wakeling and Ellington 1997). Nevertheless, our results suggest that for at least some members of the Zygoptera, wings are not exactly identical, with fore- and hindwings varying in the curvature of the wing tip (Figure 2a) and slenderness of the wing base (Figure 2b) (see also Outomuro and Johansson 2011). Since the wing base of the forewing was found to be narrower than that of the hindwing, wing shape evolution in damselflies may also have been less constrained than previously thought. One possible explanation is that the differential fore- and hindwing shape has been selected to amplify the pigmented wing spot which plays a role in sexual signaling to potential female partners (Outomuro and Johansson 2011).

In conclusion, the hypothesis that winners and losers differ in wing shape could not be confirmed. Considering the highly significant differences in wing shape and size between fore- and hindwings, we suggest that future studies should avoid pooling such data when studying selection on wing morphology in *Calopteryx* damselflies.

**Table 1.**
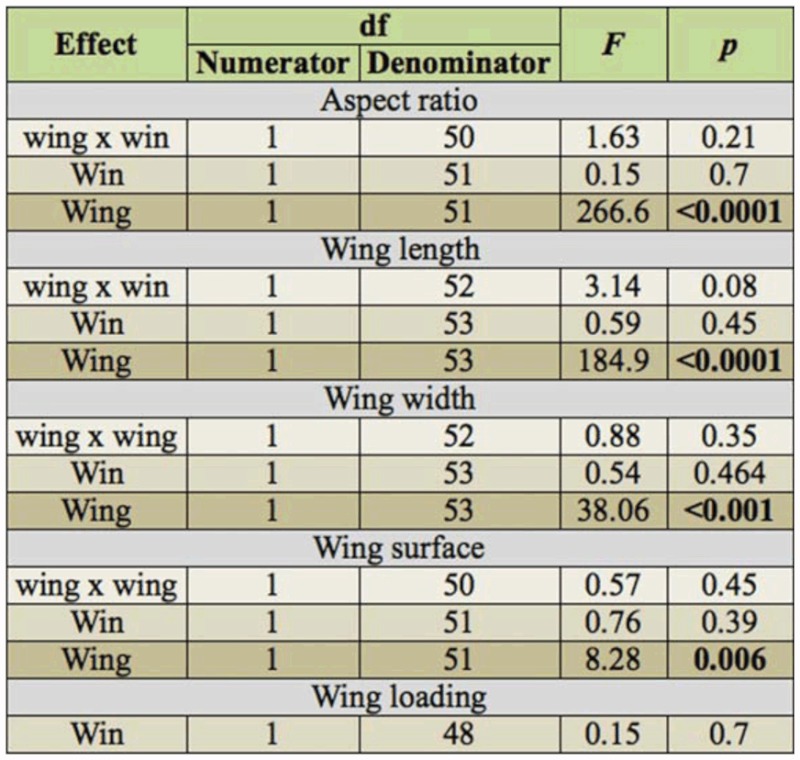
Results of the analysis of aspect ratio, wing length, wing width, wing surface, and wing loading using mixed model ANOVA. Note that wing loading is calculated using measures of both the fore- and hindwing.

**Table 2.**
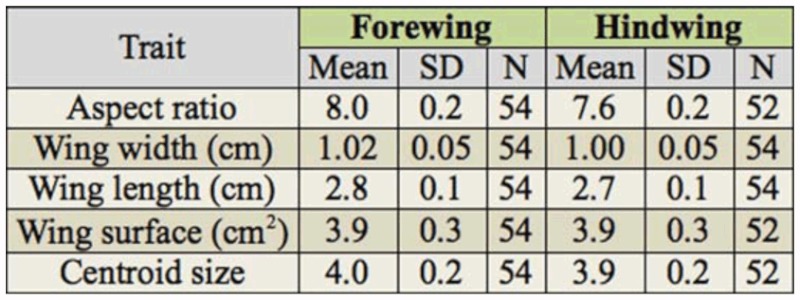
Summary of the average aspect ratio, wing length, width, surface, and centroid size for fore- and hindwing of *Calopteryx virgo*.

**Supplementary Table 1.**
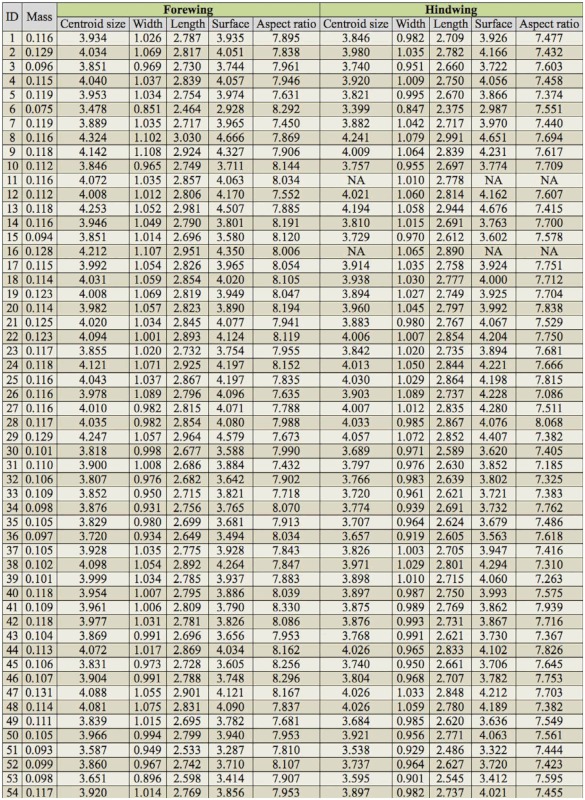
Raw data that has been collected for each *Calopteryx virgo* male using landmarks on digital images. For both fore- and hindwing, centroid size, width (cm), length (cm), surface (cm^2^), and aspect ratio are given (based on average values of left and right). Fresh mass is also denoted (g). NA = not avalailable.

## Abbreviations

RHP,: resource holding potential
